# Association of integrated biomarkers and progression-free survival prediction in patients with gastroenteropancreatic neuroendocrine tumors undergoing [177Lu]Lu-DOTA-TATE therapy

**DOI:** 10.7150/thno.112588

**Published:** 2025-05-25

**Authors:** Felix L. Herr, Christian Dascalescu, Ricarda Ebner, Moritz L. Schnitzer, Matthias P. Fabritius, Christine Schmid-Tannwald, Mathias J. Zacherl, Vera Wenter, Lena M. Unterrainer, Matthias Brendel, Adrien Holzgreve, Rudolf A. Werner, Christoph J. Auernhammer, Christine Spitzweg, Thomas Knösel, Tanja Burkard, Jens Ricke, Maurice M. Heimer, Gabriel T. Sheikh, Clemens C. Cyran

**Affiliations:** 1 Department of Radiology, LMU University Hospital, LMU Munich, Munich, Germany; 2 Department of Nuclear Medicine, LMU University Hospital, LMU Munich, Munich, Germany; 3 Bayerisches Zentrum für Krebsforschung (BZKF), partner site Munich, Germany; 4 DZNE - German Center for Neurodegenerative Diseases, Munich, Germany; 5 Munich Cluster for Systems Neurology (SyNergy), University of Munich, Munich, Germany; 6 German Cancer Consortium (DKTK), partner site Munich, a partnership between DKFZ and LMU, Germany; 7 The Russell H. Morgan Department of Radiology and Radiological Sciences, Division of Nuclear Medicine and Molecular Imaging, Johns Hopkins University School of Medicine, Baltimore, Maryland; 8 Interdisciplinary Center of Neuroendocrine Tumors of the GastroEnteroPancreatic System (GEPNET-KUM, ENETS certified Center of Excellence), LMU University Hospital, LMU Munich, Munich, Germany; 9 Department of Medicine IV, LMU University Hospital, LMU Munich, Munich, Germany; 10 Department of Pathology, LMU University Hospital, LMU Munich, Munich, Germany.

**Keywords:** GEP-NET, PRRT, histopathology, Ki-67, proliferation index, Krenning score, somatostatin receptor

## Abstract

Integrated biomarkers that predict survival in patients with gastroenteropancreatic neuroendocrine tumors (GEP-NET) receiving peptide receptor radionuclide therapy (PRRT) are still limited. This study aims to identify predictors of progression-free survival (PFS) in patients with GEP-NET undergoing two cycles of PRRT.

**Methods:** This single-center retrospective study included 178 patients with GEP-NET (G1 and G2) who received at least two consecutive cycles of PRRT with [177Lu]Lu-DOTA-TATE and underwent somatostatin receptor (SSTR)-PET/CT before and after therapy. At baseline, Krenning score (KS) > 2, clinical, pathological and laboratory parameters were collected and correlated to PFS. Survival predictors were analyzed using univariate and multivariate models. For goodness-of-fit analysis, the Akaike information criterion and Harrell concordance index were determined. To determine the impact on the regression model the Wald-Test was performed.

**Results:** In univariate analysis, KS 3 (vs. KS 4; HR, 2.02; 95% CI, 1.27-3.22; p = 0.012), Ki-67 > 5 % (HR, 2.00; 95% CI, 1.31-3.04; p = 0.008), CgA > 200 ng/mL (HR, 1.77; 95% CI, 1.14-2.76; p = 0.027) and NSE > 35 ng/mL (HR, 2.37; 95% CI, 1.44-3.89; p < 0.008) were significantly associated with shorter PFS, with CgA providing the highest C-index (0.6). In multivariate analysis , KS 3 (vs. KS 4; HR, 1.94; 95% CI, 1.17-3.21; p = 0.01), CgA > 200 ng/mL (HR, 1.76; CI, 1.08-2.87; p = 0.024), NSE > 35 ng/mL (HR, 1.98; 95% CI, 1.17-3.36; p = 0.011), and Ki-67 > 5 % (HR, 1.89; 95% CI, 1.18-3.02; p = 0.008) were significantly associated with reduced PFS. Including KS into multivariate analysis significantly improved the Cox regression model performance, as shown by a reduction in Akaike Information Criterion (592/596) and an increase in concordance index (0.66/0.65). The Wald test for individual variables supported the significance of both Ki-67 (7.1) and KS (6.7) as independent predictors of PFS.

**Conclusions:** NSE, CgA, KS and Ki-67 emerged as independent predictors of PFS in GEP-NET patients scheduled for two cycles of PRRT, thereby emphasizing the importance of integrated diagnostics including in- and ex-vivo biomarkers to identify high-risk individuals prone to disease progression.

## Introduction

Somatostatin receptors, most frequently subtype 2 (SSTR2), are overexpressed on the surface of gastroenteropancreatic neuroendocrine tumor (GEP-NET) 1,2, and can be exploited for SSTR-directed PET 3. In this regard, several radiotracers are currently available, including Gallium-68 labeled, and more recently, fluorine radiotracers, such as [18F] silicon fluoride acceptor tagged Tyr(3)octreotate ([^18^F]SiTATE) 4-9. In a theranostic setting, peptide receptor radionuclide therapy (PRRT) takes advantage of SSTR overexpression in GEP-NET patients by using ß-emitting Lutetium-177 labeled therapeutic equivalents 10. Of note, sufficient uptake on PET in sites of disease is a prerequisite 11,12 and the PET-based modified Krenning score (KS) investigating uptake in unaffected healthy parenchyma can identify patients eligible for such a systemic therapeutic approach 6,13.

Effective management of NET requires a precise diagnostic process due to the heterogeneity of these tumors. 14. According to the North American Neuroendocrine Tumor Society (NANETS) and European Neuroendocrine Tumor Society (ENETS) guidelines histopathologic biopsy should provide information regarding the histologic classification, the degree of differentiation, and the proliferation-based grading with indication of the Ki-67 index 15,16. Moreover, in accordance with the NANETS and ENETS Guidelines, circulating biomarkers, specifically chromogranin A (CgA) and neuron-specific enolase (NSE), should be collected at baseline for all NET 16,17.

The integration of imaging, pathology, clinical and laboratory testing data for cancer patients is referred to as integrated diagnostics 18. Using integrated diagnostics has the potential to improve the diagnosis and therapeutic management of numerous diseases, including cancer and it has been demonstrated that the combination of variables from multiple diagnostic disciplines is a more effective approach 18. It can be used to evaluate prognosis and facilitate therapy guidance for patients with prostate and breast cancers 19,20.

Integrated biomarkers of survival for patients with GEP-NET receiving PRRT remain limited and thus, we hypothesize that integrated biomarkers combing imaging, pathological, clinical and laboratory parameters at baseline are associated with progression-free survival (PFS) in patients with GEP-NET undergoing two cycles of PRRT. This study aims to evaluate integrated diagnostics and to identify independent potential predictors of PFS in patients with GEP-NET under two cycles of PRRT at baseline.

## Methods

### Study design

This single-center retrospective study was approved by the local ethics committee (approval number 19-027). Informed consent was waived due to the retrospective study design. All patients with histologically proven GEP-NET who underwent PRRT at an ENETS-certified center for GEP-NET at a tertiary university medical center between 2012 and 2023 were reviewed. The decision to perform PRRT was made by a multidisciplinary tumor board (MDT). The MDT defined clinical progression through a semi-quantitative and visual determination of clinically relevant tumor dynamics, reminiscent of RECIST 1.1, yet without its concrete application. The decision-making process of the MDT was based on a comprehensive evaluation of clinical and laboratory parameters, including CgA and NSE levels, CT-derived assessments of tumor size, and PET-based evaluation of SSTR expression. Key factors influencing the recommendation for PRRT included the extent of SSTR expression, primary tumor site, Ki-67 proliferation index, overall tumor burden, and observed tumor dynamics. The MDT operates as a dedicated GEP-NET tumor board within the interdisciplinary Center of Excellence for GEPNET at our tertiary care institution, officially certified by ENETS. The board meets weekly and reviews approximately 800 cases annually. It brings together experts from multiple disciplines, including endocrinology, gastroenterology, oncology, surgery, nuclear medicine, radiology, radiation oncology, and pathology, ensuring access to the most current diagnostic and therapeutic strategies.

The study included patients with differentiated NET G1 and G2, imaged with [68Ga]Ga-DOTA(0)-Phe(1)-Tyr(3))octreotate ([^68^Ga]Ga-DOTA-TATE), [68Ga]Ga-DOTA(0)-Phe(1)-Tyr(3))octreotide ([^68^Ga]Ga-DOTA-TOC), [68Ga]Ga-DOTA,1-Nal(3)]-octreotide ([^68^Ga]Ga-DOTA-NOC) and [^18^F]SiTATE PET/CT at baseline and at follow-up, and treated with at least two consecutive cycles and a maximum of six cycles of [177Lu]177Lu-DOTA-Tyr3-octreotate ([^177^Lu]Lu-DOTA-TATE) PRRT. The interval between initial diagnosis and first administration of PRRT was designated as the time since diagnosis. *N* = 20 patients underwent two cycles of PRRT only and did not receive any further PRRT cycles. The median time between baseline and the first cycle was 1.6 ± 1.2 months, whereas the interval between the initial two cycles had a median duration of 2.3 ± 0.7 months. The median time from baseline to follow-up after 2 cycles was 6.2 ± 1.5 months. During the follow-up period, *n* = 93 (52%) patients exhibited progressive disease (PD) according to the GEP-NET MDT. In total *n* = 178 patients were enrolled in this study.

### Clinical and laboratory parameters

Multiple demographic parameters such as age, initial diagnosis, primary tumor location and previous oncological therapies before PRRT were collected from electronic health archives. At baseline and follow-up, a comprehensive set of clinical, pathological and laboratory data was collected. This included body mass index (BMI), Ki-67, C-reactive protein (CRP), albumin, leukocytes, erythrocytes, hemoglobin, thrombocytes, NSE and CgA.

### Outcome measures

PFS was defined as the time between baseline and progression, as reported by a GEP-NET MDT assessment or reported death. The follow-up time was defined as the time from baseline to the loss of follow-up.

### Imaging protocol and analysis

At baseline, patients underwent SSTR-PET/CT on three different PET/CT systems (Siemens Biograph mCT, baseline* n* = 110; Siemens Biograph 64, baseline *n* = 58; GE Discovery 690 baseline *n* = 10). PET examinations within the data set were acquired with 4 different SSTR-targeting radiotracers: [^68^Ga]Ga-DOTA-TATE (baseline *n* = 71), [^68^Ga]Ga-DOTA-TOC (baseline *n* = 70), [^68^Ga]Ga-DOTA-NOC (baseline *n* = 1) and [^18^F]SiTATE (baseline *n* = 36). Mean applied activity of ^68^Ga- and ^18^F-tracer was respectively 215 ± 44 MBq and 232 ± 32 MBq at baseline. PET was acquired 60-90 minutes following the injection of the radiotracer. PET/CT scans were performed with a diagnostic CT scan of the neck, thorax, abdomen and pelvis (100-190 mAs, 120 kV, collimation 2 × 5mm, pitch of 1.5). Images from the Biograph mCT20 (Siemens Healthineers, Erlangen, Germany) were reconstructed using the reconstruction algorithm TrueX with 2 iterations (21 subsets) and time of flight (TOF) on a 200 x 200 matrix, resulting in a voxel size of 2mm x 4mm x 4mm. A Gaussian filter with a 3mm Full width at half maximum (FWHM) was applied to the reconstructed images from the Biograph mCT20. Images from the Biograph 64 TruePoint (Siemens Healthineers, Erlangen, Germany) were reconstructed using the reconstruction algorithm TrueX with 3 iterations (21 subsets) on a 168 x 168 matrix, resulting in a voxel size of 3mm x 4mm x 4mm. A Gaussian filter with a 3mm FWHM was applied to the reconstructed images from the Biograph 64 TruePoint. Images from Discovery 690 (GE Healthcare, Chicago, Illinois, USA) were reconstructed using the reconstruction algorithm VPFX with 3 iterations (21 subsets) on a 256 x 256 matrix, resulting in a voxel size of 3mm x 4mm x 4mm. A Gaussian filter with a 6.5mm FWHM was applied to the reconstructed images from Discovery 690.

PET/CT studies were assessed independently by two board-certified hybrid imaging experts (CCC, MPF) with 20- and 5-years' experience in hybrid imaging. SSTR expression of tumor lesions was visually assessed by the KS based on the lesion with the highest uptake in each patient 21. Patients exhibiting a KS > 2 received PRRT 22.

### Statistical analysis

The statistical programming tool R (version 4.3.0, R Foundation for Statistical Computing, Vienna, Austria) was used for data analysis. The threshold for statistical significance was set at p < 0.05. To evaluate data distribution Kolmogorov-Smirnov tests were performed, and parametric or non-parametric tests were performed accordingly. To address missing data, multiple imputations were performed using the R package mice. Assuming missing at random, five imputed datasets were generated using predictive mean matching, which is suitable for continuous variables such as CRP. Each imputed dataset was analyzed separately, and the pooled results were calculated using Rubin's rules. Sensitivity analyses were conducted to compare outcomes based on imputed versus non-imputed data. As the results remained consistent across approaches, the final regression models were based on the original non-imputed dataset, excluding cases with missing values in the respective variables. Survival predictors were analyzed using both univariate and multivariate models. To identify optimal prognostic thresholds, we performed a data-driven cut-point analysis using the survminer package in R. This method applies maximally selected rank statistics to determine the cut-off value that best separates PFS outcomes, based on the log-rank test. The resulting cut-off values were chosen as they yielded the greatest discriminatory power in our cohort. These values were subsequently used for both univariate and multivariate survival analyses. To account for multiple comparisons in the univariate survival analyses, p-values were adjusted using the Benjamini-Hochberg procedure for false discovery rate control, as implemented in the stats package in R. This method is appropriate for settings where multiple independent hypotheses are tested, as in univariate models evaluating several variables separately. No p-value adjustment was applied to the multivariate Cox regression models, as all covariates are analyzed simultaneously within a single model, and multiple testing correction is generally not standard in this context. Kaplan-Meier survival curves were generated for univariate analysis, and differences in survival between groups were compared using the log-rank test. Multivariate analysis was conducted using Cox proportional hazards regression to identify independent predictors of PFS by the stepwise model with backward elimination. The variables included in the multivariate analysis were those found to be significant in the univariate analysis, alongside clinically relevant parameters. For goodness-of-fit analysis, the Akaike Information Criterion and Harrell concordance index were also calculated to compare different Cox regression models with each other and with a null model (without any parameter). In this regard, a lower Akaike information criterion and a higher Harrell concordance value indicate a better-fit model 23,24. Deviance analysis (ANOVA) was performed to assess model improvement when specific variables were added to the regression model. To assess statistical significance of the variables in the Cox regression model, the Wald test was applied. All statistical analyses were reviewed and validated by the department's statistician, TB, MSc, to ensure methodological accuracy and robustness.

## Results

### Patient characteristics

The patient cohort flow chart is presented in Table 1. The mean follow-up period was 41 ± 18 months (range, 7 - 124 months) in a cohort with a mean age at baseline of 63.5 ± 10.6 years (range, 35 - 88 months). The primary tumors were located in the small bowel (*n* = 98/178, 55%), pancreas (*n* = 37/178, 21%), cancer of unknown primary (*n* = 21/178, 12%), ileocecal junction (*n* = 11/178, 6%), large bowel/rectum (*n* = 9/178, 5%), and other locations (*n* = 2/178, 1%). According to baseline SSTR-PET/CT, the metastatic sites identified were the liver (*n* = 144/178, 81%), lymph nodes (*n* = 108/178, 61%), mesenterial/peritoneal (*n* = 108/178, 61%), bone (*n* = 105/178, 59%), and other locations (*n* = 24/178, 13%). Median progression-free survival of all patients was 37.7 ± 18 months (range, 4 - 105 months). The 1-, 2-, 3- and 5-year progression-free survival of all patients was 92%, 69%, 52% and 31%, respectively. Among all patients, *n* = 152/178 (85%) patients received the planned maximum of four PRRT cycles. *N* = 26 patients received only two cycles of PRRT due to tumor progression (*n* = 8/26, 31%), mixed response (*n* = 3/26, 11%), reduced SSTR-expression (*n* = 2/26, 8%), adverse events (*n* = 13/26, 46%) and patient will (*n* = 1/26, 4%).

### Clinical and laboratory parameters

An overview of the clinical and laboratory parameters at baseline and follow-up is presented in Table 2. In less than *n* = 10 patients CgA has not been evaluated at baseline or follow-up. CRP showed the highest number of missing entries (*n* = 51 at baseline, *n* = 38 at follow-up), followed by NSE (*n* = 30 at baseline and *n* = 6 at follow-up) and albumin (*n* = 30 at baseline and *n* = 1 at follow-up).

PRRT led to a decrease in leukocytes, erythrocytes, hemoglobin and thrombocytes (p < 0.001) from baseline to follow-up. Including all patients, NSE showed a significant decline from baseline to follow-up (p = 0.015), whereas CgA showed a non-significant decrease (p = 0.053). Patients who did not experience any progression on follow-up showed a significantly lower NSE at baseline and follow-up (p < 0.001), whereas no correlation between CgA at any time point and progression on follow-up could be determined.

### PFS Analysis under PRRT

**Figure 1:**
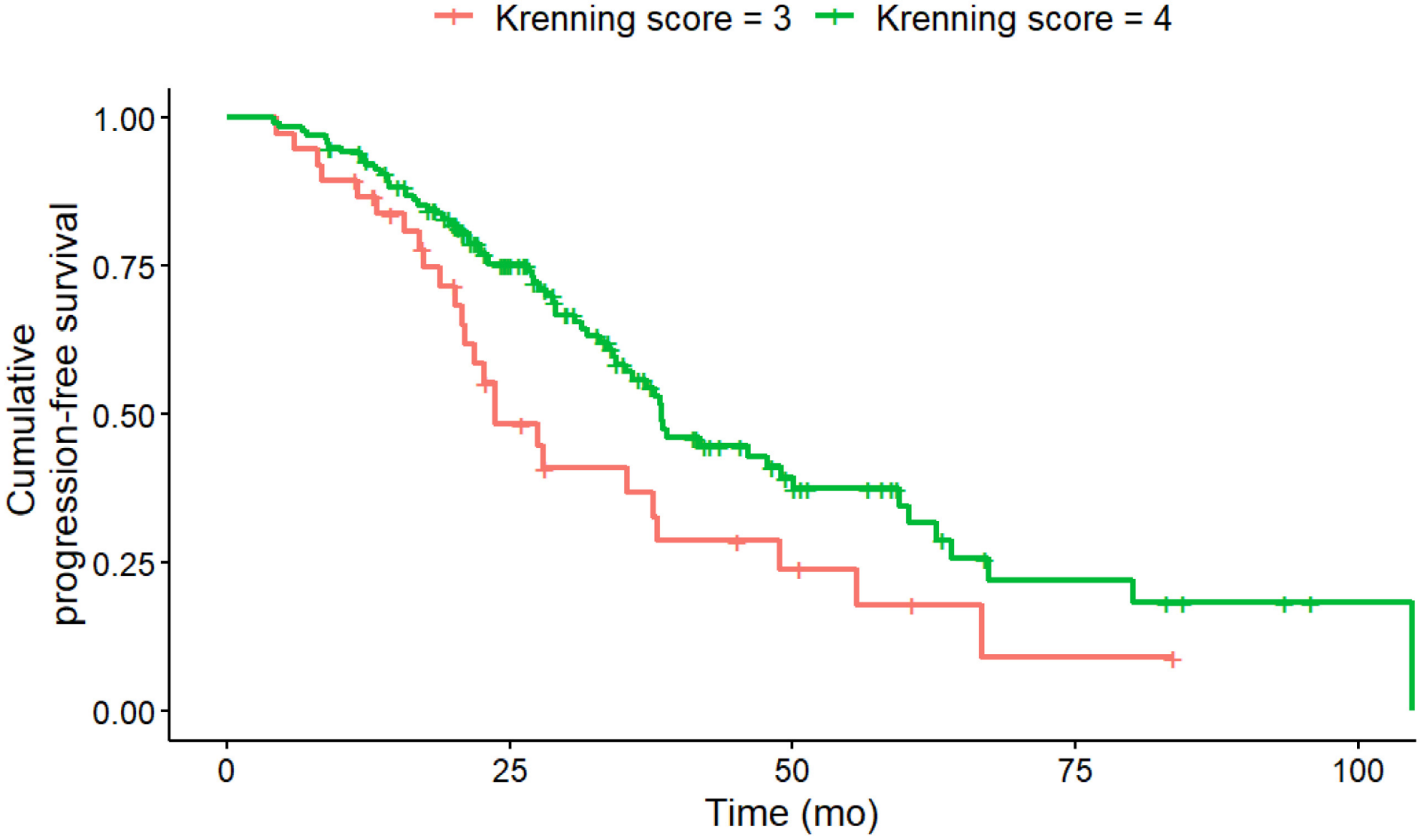
PFS according to KS at baseline. Baseline KS 3 (n = 38) was associated with a significantly (HR, 2.02; 95% CI, 1.27-3.22; p = 0.012) shorter median PFS (KS 3: 23.7; KS 4: 38.4 months) compared to KS 4 (n = 140).

**Figure 2:**
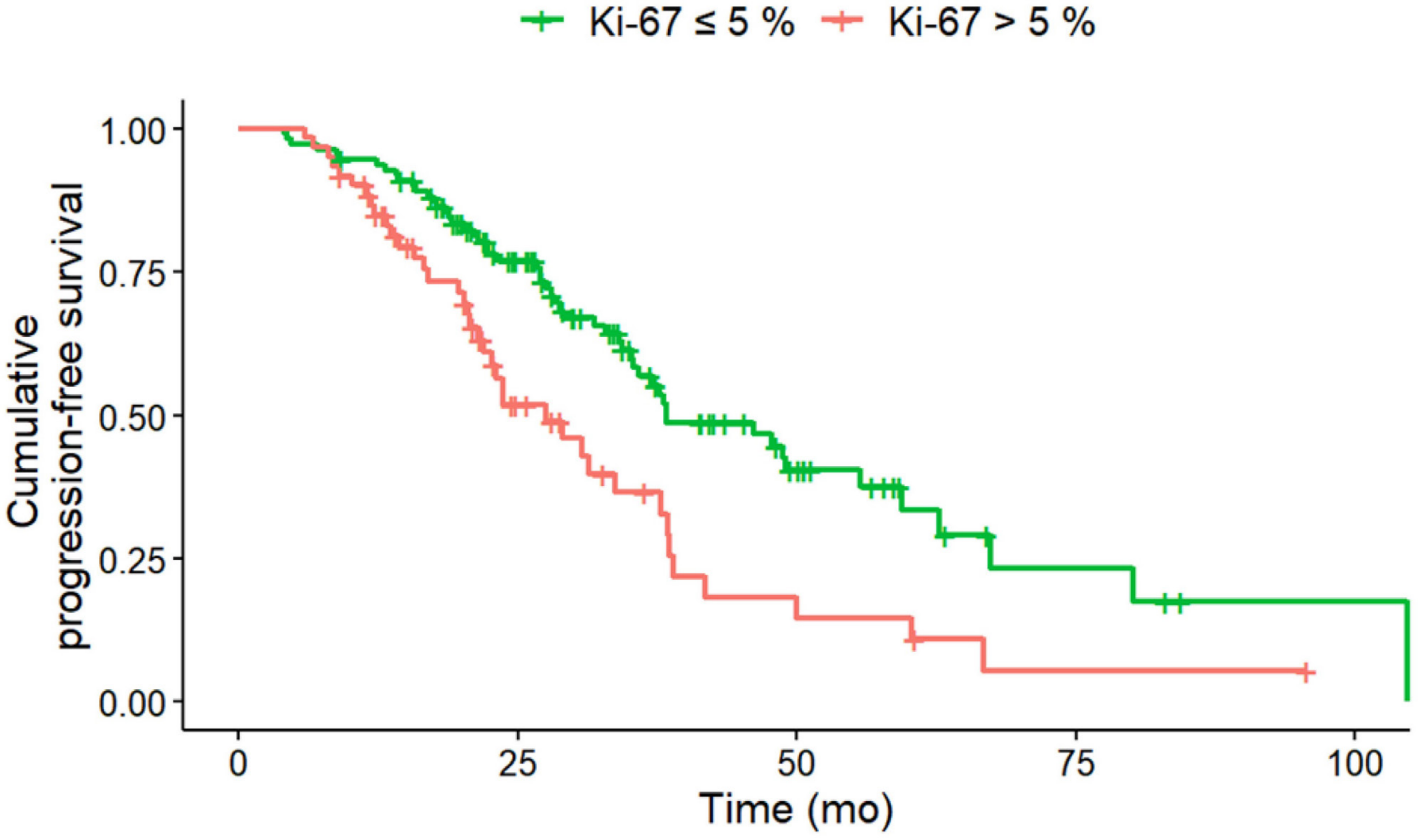
PFS according to Ki-67 at baseline. Baseline Ki-67 index of greater than 5% (n = 60) was associated with a significantly (HR, 2.00; 95% CI, 1.31-3.04; p = 0.008) shorter median PFS (Ki-67 > 5% 27.5 months; Ki-67 ≤ 5%: 38.4 months) compared to Ki-67 index of less than or equal to 5% (n = 111).

**Figure 3:**
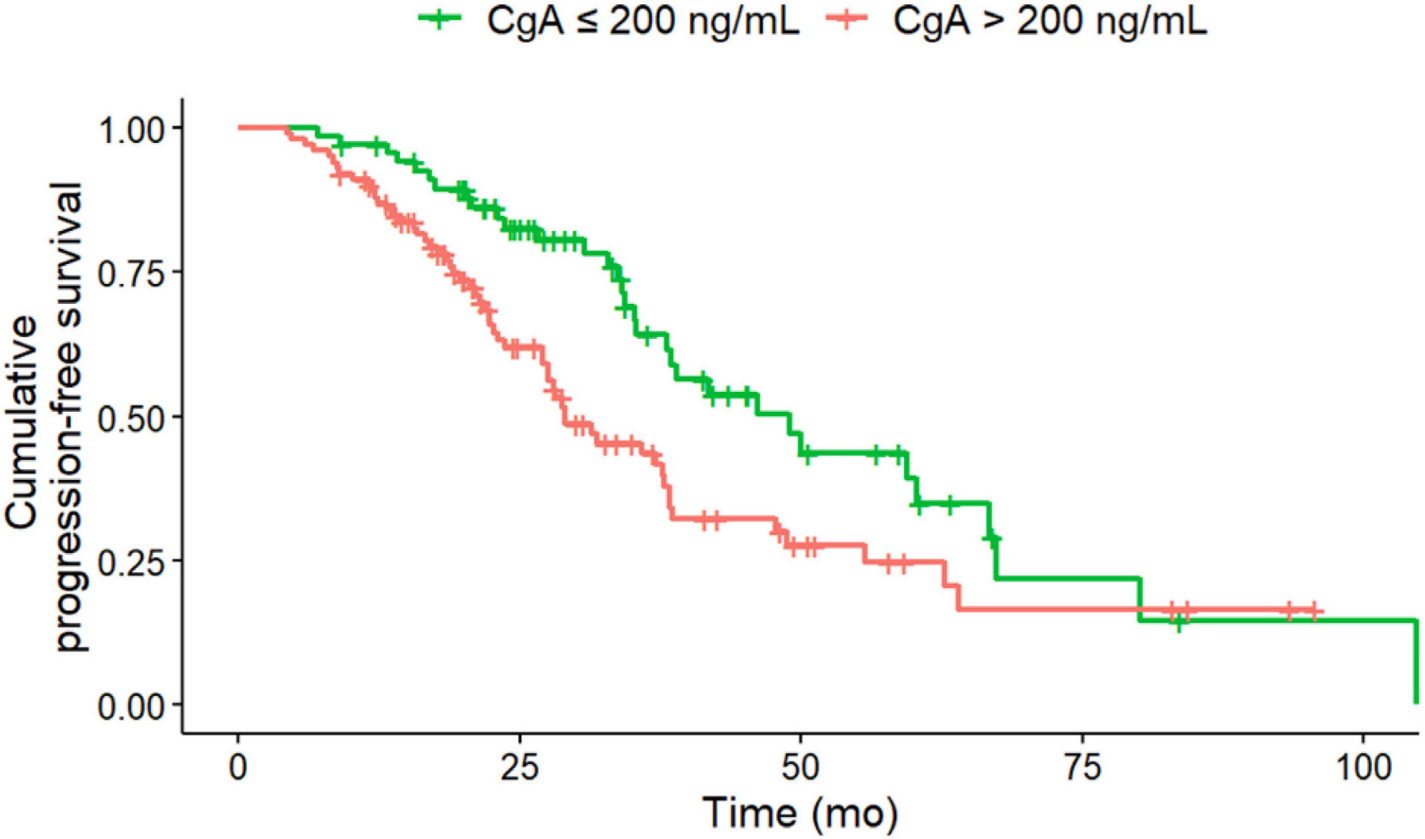
PFS according to CgA at baseline. Baseline CgA of greater than 200 ng/mL (n = 100) was associated with a significantly (HR, 1.77; 95% CI, 1.14-2.76; p = 0.011) shorter median PFS (CgA > 200 ng/ml 29 months; CgA ≤ 200 ng/ml: 46.1 months) compared to CgA of less than or equal to 200 ng/mL (n = 68).

**Figure 4:**
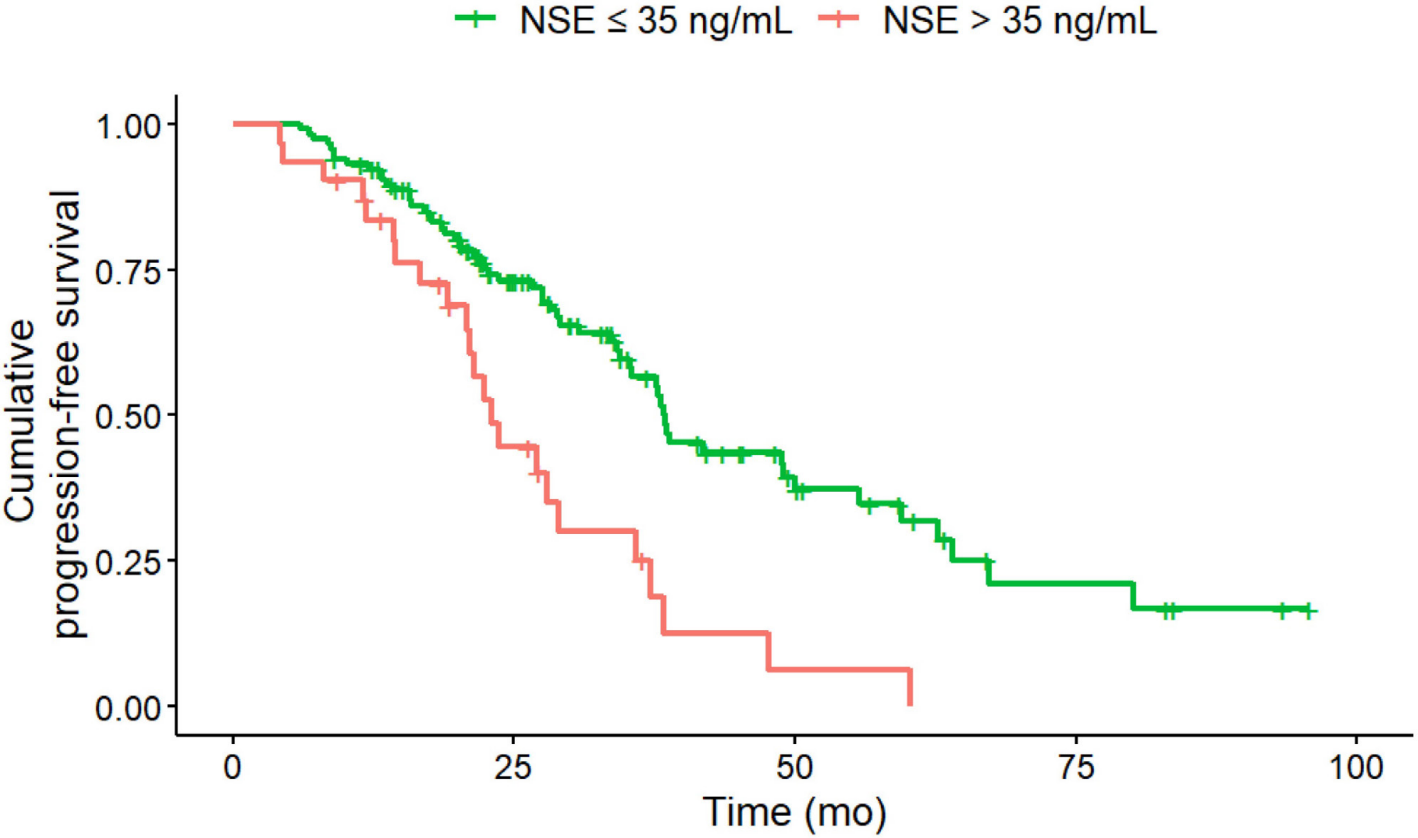
Baseline NSE of greater than 35 ng/mL (n = 52) was associated with a significantly (HR, 2.37; 95% CI, 1.44-3.89; p = 0.008) shorter median PFS (NSE > 35 ng/ml: 23 months; NSE ≤ 35 ng/ml: 38.1 months) compared to NSE of less than or equal to 35 ng/mL (n = 96).

In univariate analysis (log-rank-test), several baseline factors were significantly associated with shorter PFS (Table 3). Patients with KS 3 (vs. KS 4; HR, 2.02; 95% CI, 1.27-3.22; p = 0.012), CgA > 200 ng/mL (HR, 1.77; 95% CI, 1.14-2.76; p = 0.027), NSE > 35 ng/mL (HR, 2.37; 95% CI, 1.44-3.89; p = 0.008) and Ki-67 index > 5 % (HR, 2.00; 95% CI, 1.31-3.04; p = 0.008) had significantly shorter PFS (Figure 1 - 4). Additionally, the following parameters were significantly correlated with shorter PFS: primary tumor in the pancreas (HR, 1.70; 95% CI, 1.04-2.78; p = 0.047), erythrocytes ≤ 4 million/ µl (HR, 1.87; 95% CI, 1.07-3.28; p = 0.043), hemoglobin ≤ 12 g/dl (HR, 2.02; 95% CI, 1.13-3.62; p = 0.033), CRP > 0.5 mg/ dL (HR, 2.15; 95% CI, 1.20-3.83; p = 0.027) and albumin < 4.1 g/ dL (HR, 1.76; 95% CI, 1.1-2.83; p = 0.033). However, BMI, leukocytes and thrombocytes were not significant (p ≥ 0.11).

In the multivariate Cox regression analysis at baseline CgA > 200 ng/mL (HR, 1.76; CI, 1.08-2.87; p = 0.024), NSE levels > 35 ng/mL (HR, 1.98; 95% CI, 1.17-3.36; p = 0.011), a Ki-67 index > 5% (HR, 1.89; 95% CI, 1.18-3.02; p = 0.008) and KS 3 (vs. KS 4; HR, 1.94; 95% CI, 1.17-3.21; p = 0.01) were associated with shorter PFS (Table 3). Only the Ki-67 index showed a higher z-score than the KS at baseline, indicative for improved predictive value.

The inclusion of KS into the multivariate analysis model significantly improved the Cox regression model performance, as shown by a reduction in Akaike Information Criterion values (592/596) and an increase in the concordance index (0.66/0.65). Deviance analysis confirmed the value of the KS, with a significant p-value (p = 0.03). The Wald test for individual variables supported the significance of both the Ki-67 index (7.1) and KS (6.7) as independent predictors of PFS.

## Discussion

In the present study including 178 GEP-NET patients scheduled for PRRT, we identified in-vivo image-based (KS) and ex-vivo (Ki-67, CgA and NSE) biomarkers as independent predictors to identify patients prone to disease progression, thereby supporting our hypothesis of an integrated imaging, laboratory and histopathological approach for outcome prediction. Of note, we focused on patients scheduled for two cycles of PRRT and thus, the herein identified items may serve as valuable tools to determine high-risk individuals already at an early therapeutic stage.

### Clinical and laboratory parameters under PRRT

A significant decrease in NSE levels was evident in patients undergoing two cycles of PRRT between baseline and follow-up. Patients who demonstrated no progress during the follow-up period exhibited significantly lower NSE levels at both time points. This indicates that NSE may serve as a promising circulating serum biomarker for monitoring the response to two cycles of PRRT. The potential of NSE as a predictive biomarker for metastatic GEP-NET following two cycles of PRRT has already been discussed. Ezziddin et al. 25 asserted that a baseline plasma level of NSE > 15 ng/mL was an independent predictor of shorter overall survival. Fuksiewicz et al. 26 demonstrated an association between NSE concentrations and clinical status, confirming the usefulness of NSE in patient monitoring and as a potential predictive indicator for PFS in patients with NENs. In our study CgA exhibited a non-significant decline under two cycles of PRRT, and no correlation could be established between CgA at any time point and progression in follow-up. A number of studies have already demonstrated the predictive role of CgA in monitoring disease progression and in assessing the response to therapy. Sabet et al. 27 reported that an elevated plasma concentration of CgA > 600 ng/mL was associated with an earlier onset of tumor progression. Moreover, patients who presented with carcinoid symptoms exhibited a shorter PFS following two cycles of PRRT 27. In the NETTER-1 trial focusing on four PRRT cycles, circulating biomarkers such as CgA and NSE were unable to predict response to PRRT or survival 28. Our results, however, support the use of NSE but not CgA as a serum biomarker of therapy response already after two cycles of PRRT.

### Integrated diagnostics for PFS prediction under PRRT

The most common contemporary application of the KS is to assess candidacy for PRRT, typically with a score greater than 2. In our study, a KS 4 was associated with a significantly longer PFS than a KS 3 at baseline in univariate and multivariate analysis. This indicates that patients with an increased in-vivo SSTR expression benefit more from PRRT, which has already been shown on a quantitative assessment 29. In both univariate and multivariate analysis, CgA levels > 200 ng/dL and NSE levels > 35 ng/dL at baseline were associated with a shorter PFS. This finding has also been reported in phase II study of Everolimus in GEP-NET, as it has been demonstrated that higher baseline levels of CgA are associated with shorter PFS. Patients with the shortest PFS exhibited elevated concentrations of both CgA and NSE at baseline 30. With regard to OS, a baseline NSE level > 15 ng/mL was identified as a predictor of reduced survival under PRRT in G1/G2 NET 31. In our study, at uni- and multivariate analysis, a Ki-67 index > 5% at baseline was found to be associated with a shorter PFS after two therapeutic cycles. This finding aligns with the observations reported by Ezziddin et al. 31, who demonstrated that a Ki-67 index greater than 10% was associated with reduced PFS and OS. There is a relevant impact of Ki-67 heterogeneity, especially in NET with high proliferation rates or in cases where imaging and histology show discordant findings, such as mismatches in PET imaging. Ki-67 heterogeneity refers to variations in proliferative activity within different tumor regions, which can significantly affect both prognostic evaluation and therapeutic response 32,33. These intratumoral differences complicate treatment planning and outcome prediction, particularly in patients considered for targeted therapies like PRRT 34. Importantly, biopsies often reflect only a small tumor area, potentially under- or overestimating the true proliferative activity. This underlines the importance of recognizing Ki-67 variability when selecting and evaluating patients for PRRT. In univariate analysis, the primary tumor location in the pancreas was associated with higher risk for disease progression, which corroborates the findings of Xu et al. 35. Patients with GEP-NET of the pancreas exhibited the poorest median OS. In the univariate analysis, an erythrocyte level of ≤ 4 million/µL and hemoglobin ≤ 12 g/dl at baseline were also associated with a shorter PFS. However, Halperin et al. demonstrated that a reduction in red blood cell mass, hemoglobin, and/or hematocrit levels are all associated with an unfavorable prognosis in patients with pancreatic NET 36. As observed by Komaç et al. 37 elevated CRP, particularly if it was > 20 mg/dL, was associated with disease progression. This finding was corroborated by our study, which demonstrated that CRP levels > 1 mg/dL at baseline were associated with a shorter PFS in univariate analysis.

In the multivariate analysis, the Ki-67 index demonstrated the highest predictive value for PFS followed by the KS. Including the latter metric in the multivariate model significantly improved the Cox regression model performance and deviance analysis confirmed the importance of the KS. The Wald test for individual variables supported the significance of both, the Ki-67 index and KS, as independent predictors of PFS. Tao et al. reported already that the overall prognosis of GEP-NET patients showed a decreasing trend with the increase of Ki-67, which confirmed the significance of Ki-67 index as a prognostic marker for the prognosis of GEP-NET 38. In addition, our study shows that evaluation of the KS at baseline has similar importance for predicting PFS as the Ki-67 index, CgA and NSE. Therefore, the molecular imaging score of Krenning should be employed in conjunction with histopathological (Ki-67) and biochemical (NSE, CgA) biomarkers at baseline for the integrated diagnosis of PFS prediction in GEP-NET under two cycles of PRRT. Of note, all four parameters are easily obtained and are part of the standard diagnostic work-up in GEP-NET patients. Our multivariate Cox regression model, which integrates biochemical markers (CgA, NSE), histopathological data (Ki-67 index), and molecular imaging (KS), reflects an integrated diagnostic approach to prognostic assessment in PRRT-treated NET patients. The combination of these multimodal parameters improved the overall model performance, as indicated by a reduction in AIC and an increase in the C-index. This finding supports the concept of integrated diagnostics by demonstrating how the inclusion of complementary clinical, molecular, and imaging data can enhance prognostic accuracy beyond single-modality assessments. Moreover, the combination of multiple biomarkers may enhance the prediction of PFS by capturing complementary prognostic signals. Integrative approaches that combine histopathological markers, functional imaging parameters, and circulating biomarkers have shown to provide a more comprehensive prognostic assessment 39. This allows for improved patient stratification, enabling more personalized and effective selection of candidates for PRRT 40. Combining biomarkers can help overcome the limitations of each individual marker, especially in cases where one parameter alone offers limited predictive value. For example, combining SSR expression with Ki-67 improves the ability to differentiate between indolent and aggressive disease courses. Finally, the predictive relevance of biomarkers like Ki-67 and KS specifically in the context of PRRT-treated patients highlights their added value beyond general prognostication in NETs. These biomarkers are associated with tumor radiosensitivity and can support the identification of patients most likely to benefit from PRRT 41,42. Unlike their broader prognostic role, their predictive power in PRRT settings contributes uniquely to guiding clinical decisions aimed at maximizing treatment response and disease control 43. In this context, high somatostatin receptor expression indicates a higher likelihood of effective radionuclide binding, while lower Ki-67 values are associated with slower tumor progression. The integration of both biomarkers provides a practical framework to tailor PRRT to patients most likely to achieve durable responses.

Our findings are in line with previous studies investigating biomarker-based prediction of PRRT outcomes. Multiple recent studies highlight the Ki-67 proliferation index as a key predictor of PRRT efficacy, showing that patients with lower Ki-67 (e.g. ≤~50%) achieve significantly longer median PFS and OS after PRRT than those with higher proliferative rates​ 44 Likewise, imaging-based measures of SSR expression such as the KS correlate strongly with treatment response, as higher uptake scores have been associated with greater tumor remission rates and extended PFS compared to lower scores​ 45. In terms of circulating biomarkers, baseline CgA has been linked to tumor burden and poorer PRRT outcomes - for example, one analysis identified elevated CgA as an adverse prognostic factor (combined with tumor volume, HR ~2.7 for 5-year OS) and determined a high cutoff (~1250 μg/L) that predicted significantly lower survival​ 46. NSE has also emerged as a potential predictor in limited data: in one cohort of 74 PRRT-treated NET patients, a baseline NSE >15 ng/mL independently predicted shorter post-PRRT overall survival (hazard ratio ~2.2, *p* = 0.035)​ 45. Overall, these independent reports are consistent with our findings and support the growing consensus that a low proliferative index, strong somatostatin receptor expression, and favorable biochemical profiles are associated with improved outcomes under PRRT. Furthermore, our retrospective analysis in a detailed-characterized cohort of 178 patients demonstrates that an integrated diagnostic approach—combining imaging, biochemical, and histopathological parameters—can effectively predict PFS in GEP-NET patients undergoing PRRT. Further studies may also evaluate other PET-based metrics, such as standardized uptake value or SSTR-positive tumor volume. Nonetheless, the KS as a visual assessment tool on a four-point scale is easy to implement in clinical practice, thereby avoiding the strenuous process of whole-body PET segmentation.

### Limitations

The primary limitations of this study are its retrospective design, limited follow-up time, and single-center assessment. As this study included only patients with GEP-NET (G1 and G2) treated with at least two consecutive cycles of PRRT, it should be noted that some patients underwent even more cycles and thus, future analyses should also assess predictive parameters at baseline after four PRRT cycles. RECIST 1.1 offer a standardized approach for assessing tumor response to therapy based on changes in lesion size and are currently endorsed by the ENETS for evaluating PRRT response 47. However, given the often indolent course of NETs and the frequent occurrence of metastatic dedifferentiation over time, relying solely on morphological changes may underestimate the actual therapeutic effect 6,48. Consequently, RECIST 1.1 shows limited sensitivity in capturing treatment response in NETs. In this study, PFS was derived from the MDT decision, which defined clinical progression based on a semi-quantitative and visual assessment of relevant tumor dynamics—conceptually similar to RECIST 1.1, though not applied in a strict or formalized manner. Another limitation of this study is the use of different SSTR-targeting radiotracers for PET imaging, including [⁶⁸Ga]Ga-DOTA-TATE, [⁶⁸Ga]Ga-DOTA-TOC, [⁶⁸Ga]Ga-DOTA-NOC, and [¹⁸F]SiTATE. While these tracers have shown comparable diagnostic accuracy in clinical practice, minor differences in receptor subtype affinity and biodistribution may influence image interpretation. However, these differences are considered minimal and are unlikely to significantly affect the semiquantitative assessment of the KS, which remains comparable across tracers 4,6-9.

## Conclusions

Integrated baseline diagnostics including in-vivo PET-based (Krenning) and ex-vivo histopathological (Ki-67) and laboratory (CgA and NSE) metrics in GEP-NET patients scheduled for two cycles of PRRT have been demonstrated to enhance the accuracy of PFS prediction. As such, those routinely assessed clinical parameters may be useful to identify high-risk individuals prone to disease progression already at an early therapeutic stage.

## Figures and Tables

**Table 1:** Clinical characteristics of study population

	*n* = 178
**Male to female**	108 / 70
**Mean age at baseline** mean (SD)	63.5 years (10.6)
**Times** mean (SD)	
Mean follow-up	41 months (18)
Time since diagnosis to baseline	47 months (56)
Baseline to first cycle PRRT	1.6 months (1.2)
Baseline to follow-up	2.3 months (0.6)
**BMI (kg/m^2^)** mean (SD)	24.9 (4.3)
**Primary tumor location**, n (%)	
Small bowel	98 (55%)
Pancreas	37 (21%)
Cancer of Unknown Primary	21 (12%)
Ileocecal junction	11 (6%)
Large bowel / Rectum	9 (5%)
Other	2 (1%)
**Tumor grade**, n (%)	
NET G1 (Ki-67 ≤ 2%)	54 (30%)
NET G2 (2 < Ki-67 ≤ 20%)	119 (67%)
Unknown	5 (3%)
**Metastases**, n (%)	
Liver	144 (81%)
Lymph nodes	108 (61%)
Mesenterium / Peritoneum	108 (61%)
Bone	105 (59%)
Other	24 (13%)
**Therapy Approach before** PRRT *, n (%)	
Surgical resection of primary tumor	105 (59%)
Long-acting somatostatin analogue	110 (62%)
Local ablative liver therapies	49 (28%)
Chemotherapy or everolimus	30 (17%)
Bone-targeted radiotherapy	6 (3%)

*some patients received more than one treatment modality before PRRT. SD = standard deviation; PRRT = peptide receptor radionuclide therapy; BMI = body mass index; NET = neuroendocrine tumor.

**Table 2:** Overview of clinical and laboratory parameters at baseline and follow-up and results of the paired one-sided T-test. PRRT led to a significant decrease in blood cells from baseline to follow-up. NSE showed a significant decline from baseline to follow-up, whereas CgA showed a non-significant tendency.

Variable	Baseline Mean (SD)	Follow-up Mean (SD)	One sided T-Test (P)
Body mass index (kg/m²)	24.9 (4.2)	25 (4.2)	t = 1.04 (0.85)
CRP (mg/dL)	1 (2.9)	0.5 (0.9)	t = 1.52 (0.065)
Albumin (g/dL)	4.3 (0.4)	4.3 (0.3)	t = 0.32 (0.37)
Leukocytes (G/L)	7 (2.2)	5.1 (1.5)	t = 15.25 (< 0.001) *
Erythrocytes (million/µL)	4.5 (0.5)	4.2 (0.5)	t = 9.99 (< 0.001) *
Hemoglobin (g/dL)	13.6 (1.6)	12.9 (1.4)	t = 7.50 (< 0.001) *
Thrombocytes (g/L)	256.8 (91.4)	199.3 (64.1)	t = 10.02 (< 0.001) *
NSE (ng/mL)	30.4 (38)	23 (10.8)	t = 2.18 (0.015) *
CgA (ng/mL)	3896.4 (24084.2)	1749.2 (8888.1)	t = 1.62 (0.053)

CRP = C-reactive protein; NSE = neuron specific enolases; CgA = Chromogranin A.

**Table 3:** Univariate and multivariate analyses of factors contributing to PFS. In the univariate analysis (log-rank-test), CgA > 200 ng/mL, NSE > 35 ng/mL, Ki-67 index > 5%, primary tumor located in the pancreas, erythrocytes ≤ 4 million/µL, hemoglobin ≤ 12 g/dl, CRP levels > 1 mg/dL, and albumin < 4.1 g/dL (p < 0.05 for all) were associated with shorter PFS. In the multivariate Cox regression analysis, CgA > 200 ng/mL, NSE > 35 ng/mL and a Ki-67 index > 5% were associated with shorter PFS.

Variable	Univariate Analysis			Multivariate Analysis	
	Patients (n)	HR (95 % CI)	*P*	HR (95% CI)	*P*
All patients	178				
Body mass index					
≤ 18.5	10	2.05 (0.89 - 4.74)	0.112		
> 18.5	168				
Origin of primary tumor					
Pancreas	36	1.70 (1.04 - 2.78)	0,047*		
Other	137				
Ki-67 (%)					
> 5	60	2.00 (1.31 - 3.04)	0.008*	1.89 (1.18 - 3.02)	0.008*
≤ 5	111				
Krenning at baseline					
3	38	2.02 (1.27 - 3.22)	0.012*	1.94 (1.17 - 3.21)	0.01*
4	140				
CRP (mg/dL) at baseline					
> 0.5	43	2.15 (1.20 - 3.83)	0.027*		
≤ 0.5	81				
Albumin (g/dL) at baseline					
≥ 4.1	103	1.76 (1.10 - 2.83)	0.033*		
< 4.1	49				
Leukocytes (G/L) at baseline					
<5	24	1.1 (0.58 - 2.07)	0.796		
≥ 5	154				
Erythrocytes (million/µL) at baseline					
< 4	25	1.87 (1.07 - 3.28)	0.043*		
≥ 4	148				
Hemoglobin (g/dL) at baseline					
< 12	25	2.02 (1.13 - 3.62)	0.033*		
≥ 12	148				
Thrombocytes (g/L)					
< 200	49	1.06 (0.67 - 1.69)	0.796		
≥ 200	129				
NSE at baseline (ng/mL)					
> 35	52	2.37 (1.44 - 3.89)	0.008*	1.98 (1.17 - 3.36)	0.011*
≤ 35	96				
CgA at baseline (ng/mL)					
> 200	100	1.77 (1.14 - 2.76)	0.027*	1.76 (1.08 - 2.87)	0.024*
≤ 200	68				

CRP = C-reactive protein; NSE = neuron specific enolases; CgA = Chromogranin A

## Abbreviations

[18F]FDG: 2-[18F]fluoro-2-deoxy-D-glucose
[18F]SiTATE: [18F] silicon fluoride acceptor tagged Tyr(3)octreotate
[68Ga]Ga-DOTA-TATE: [68Ga]Ga-DOTA(0)-Phe(1)-Tyr(3))octreotate
[68Ga]Ga-DOTA-TOC: [68Ga]Ga-DOTA(0)-Phe(1)-Tyr(3))octreotide
[68Ga]Ga-DOTA-NOC: [68Ga]Ga-DOTA,1-Nal(3)]-octreotide
[177Lu]Lu-DOTA-TATE: [177Lu]177Lu-DOTA-Tyr3-octreotate
CgA: Chromogranin A
CRP: C-reactive protein
CT: Computed tomography
ENETS: European Neuroendocrine Tumor Society
FWHM: Full width at half maximum
GEP-NET: Gastroenteropancreatic neuroendocrine tumor
MDT: Multidisciplinary tumor board
NANETS: North American Neuroendocrine Tumor Society
NSE: Neuron-specific enolase
PET: Positron emission tomography
PFS: Progression-free survival
RECIST: Response Evaluation Criteria In Solid Tumors
PRRT: Peptide Receptor Radionuclide Therapy
SSTR: Somatostatin receptor
